# Insights into toxin-antitoxin systems in the genus *Bifidobacterium*

**DOI:** 10.1128/aem.01934-25

**Published:** 2025-11-05

**Authors:** Claudia Lefimil, Paula Bustamante

**Affiliations:** 1 Instituto de Investigación en Ciencias Odontológicas, Facultad de Odontología, Universidad de Chile 69748 , Santiago, Chile; 2 Molecular and Cellular Microbiology Laboratory, Instituto de Ciencias Biomédicas, Centro de Investigación e Innovación, Universidad Autónoma de Chile, Santiago, Chile; The Pennsylvania State University, University Park, Pennsylvania, USA

**Keywords:** toxin-antitoxin system, MazEF, RelBE, *Bifidobacterium*, probiotic, gut microbiome

## Abstract

Toxin-antitoxin (TA) systems are widespread genetic modules in prokaryotes, implicated in diverse functions including stress adaptation, genome stability, and virulence. While extensively studied in pathogenic bacteria, their presence and roles in beneficial gut microbes like *Bifidobacterium* remain underexplored. This review consolidates current knowledge on type II TA systems within the *Bifidobacterium* genus, highlighting their diversity, genomic context, and potential functional roles. Genomic analyses reveal a predominance of MazEF and RelBE families, with other systems such as VapBC, YefM-YoeB, and PumAB also identified, albeit less frequently. Experimental validation is limited, with most studies focused on *B. longum* strains. Emerging evidence suggests that these systems may contribute to acid and osmotic stress responses and mobile genetic element maintenance. The species- and strain-specific distribution of TA loci suggests their potential utility as molecular markers for strain-level microbiome analysis. Given their multifaceted roles, further functional studies are warranted to elucidate the biological significance of TA systems in *Bifidobacterium* and their implications for gut health and probiotic efficacy.

## INTRODUCTION

Probiotic bacteria, including members of the genus *Bifidobacterium*, play a vital role in maintaining host health by modulating gut microbiota, enhancing barrier function, and influencing immune responses (1, 2). To thrive in the dynamic gut environment, these microorganisms must employ robust mechanisms for stress adaptation and survival. However, the understanding of the precise molecular strategies employed to ensure effective colonization and survival in the competitive gut ecosystem remains incomplete. In this context, the mechanisms of the toxin-antitoxin (TA) genetic systems, which play a role in bacterial adaptation to constantly changing environmental conditions (3, 4), are of particular interest.

This review aims to provide an updated overview of type II TA systems in *Bifidobacterium*, integrating current genomic and experimental findings, and discussing their potential relevance to stress tolerance, gut colonization, and probiotic functionality.

## THE *BIFIDOBACTERIUM* GENUS

Members of the genus *Bifidobacterium* are high G+C content, gram-positive bacteria belonging to the phylum Actinobacteria and represent common inhabitants of the gastrointestinal tract of mammals (5). They are heterofermentative, anaerobic bacteria with a distinctive bifid shape, which gives them their name (6). There are currently 110 species of *Bifidobacterium* in the NIH database (7), comprising species found in a variety of ecological environments, including sewage, fermented or raw milk, the hindguts of birds and some insects, human blood, the oral cavity, and the gastrointestinal tracts of humans and other mammals (8, 9).

**Fig 1:**
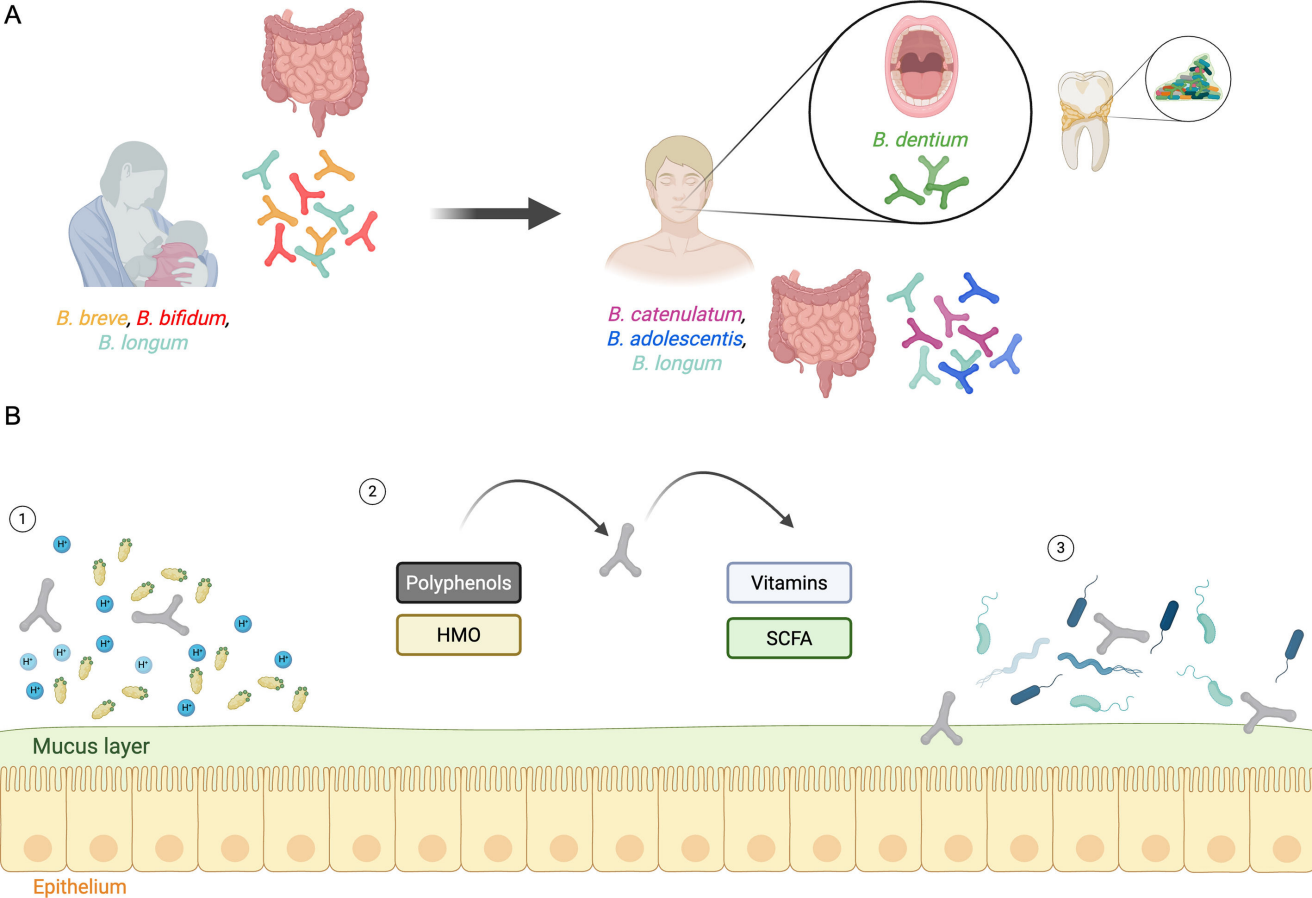
Schematic overview of the lifestyle and functional roles of *Bifidobacterium* in the human gut. (**A**) *Bifidobacterium* diversity changes during the life cycle. *Bifidobacterium* species (Y-shape entities), such as *B. longum, B. breve*, and *B. bifidum*, are transmitted from mother to infant and colonize the intestinal epithelium, particularly in early life. *B. catenulatum, B. adolescentis,* and *B. longum* are the most prevalent species in adulthood. *B. dentium* colonizes the oral cavity and has been related to caries lesions, through biofilm structure formation. (**B**) Relevant biological activities of *Bifidobacterium* to the human gut. (1) They exhibit acid and bile tolerance, supporting their persistence in the gastrointestinal environment. (2) These bacteria metabolize dietary carbohydrates, including human milk oligosaccharides and dietary polyphenols. They produce important metabolites such as vitamins and short-chain fatty acids (SCFAs) like acetate, lactate, and formate. (3) *Bifidobacterium* plays a barrier role through the competitive exclusion of pathogenic microorganisms and modulates host immunity by interacting with immune cells at the mucosal surface. Figure created with BioRender.com.

*Bifidobacterium* is a relevant genus of human gut commensals, and its communities in this ecological niche comprise diverse species, which vary with age (9). They are mostly prominent in the early-life microbiome, but these levels decrease significantly during adulthood and remain relatively stable, with another decline observed in old age (9, 10). Different species are not exclusive to a specific host age but differ in prevalence and/or abundance. *B. breve*, *B. longum*, and *B. bifidum* are generally dominant in infants, whereas *B. catenulatum*, *B. adolescentis*, and *B. longum* are prevalent in adults (9, 11–13). Of note, *B. longum* persists throughout all life stages (Fig. 1A).

This genus is often associated with host health-promoting effects, as it plays an essential role in the barrier effect and the stimulation of the immune system (5). The evidence indicates that *Bifidobacterium* depletion in the human gut microbiota is linked to various metabolic, immune, and intestinal diseases (14). Also, *Bifidobacterium* produces important metabolites (12), including vitamins, conjugated linoleic acids, and short-chain fatty acids, that benefit epithelial host cells and gut microorganisms (Fig. 1B). Consequently, some strains of *Bifidobacterium* are commonly used as probiotics (12, 15).

On the other hand, among members of this genus, the *B. dentium* species has been usually associated with pathogenicity, as in the human oral cavity, it is associated with the development of dental biofilm and caries lesions and might transiently pass the human intestine (16).

### Virulence and stress response in *Bifidobacterium*

A genome analysis of *Bifidobacterium* species revealed that 13.7% of their genes are involved in carbohydrate metabolism (17), emphasizing the importance of this function within the genus. It possesses an extensive battery of enzymes to metabolize carbohydrates, among the largest compared to other gut commensals (18).

*Bifidobacterium* faces several detrimental factors that may compromise cell viability and functionality, including exposure to oxygen or oxygen-derived free radicals, organic and bile acids, and osmotic stress. To adapt to these challenging conditions, these microorganisms have developed specific responses involving the regulated expression of various enzymes and proteins, including molecular chaperones, bile efflux transporters ATPase-associated, bile salt hydrolases, and two-component systems. All of them can effectively respond to the diverse challenges in the gut environment (9).

To deal with oxidative stress when exposed to aerobic conditions, *B. longum* NCC2705 produces alkyl hydroperoxide reductase and chaperone proteins (19). Meanwhile, strains of *B. animalis* subsp. *lactis* increase the expression of genes that encode thioredoxin peroxidases and NADH oxidase (20).

Also, the *Bifidobacterium* species have developed acid resistance mechanisms to withstand acidic conditions, through activating an F0F1-ATPase, which helps to maintain pH homeostasis by actively pumping protons out of the cell (21). Additionally, members of the genus *Bifidobacterium* may enhance their acid resistance by altering the fatty acid composition of their cell membranes (22). In addition, biofilm formation is a crucial adaptive strategy in response to acid and bile stress, relying on a multi-factorial process involving exopolysaccharide production and protein and extracellular DNA release, as observed in the *B. breve* UCC2003 (23). While these mechanisms help *Bifidobacterium* survive in the gastrointestinal environment, in *B. dentium*, they may contribute to its ability to colonize and persist in dental biofilms, potentially promoting pathogenicity (24).

### Mobile genetic elements (MGEs) in *Bifidobacterium*

The genomes of *Bifidobacterium* species vary in size, ranging from 1.73 to 3.25 Mb, with a G+C content that ranges from 52.84% to 65.53% (17). The mobilome—all MGEs within the genome—identified in *Bifidobacterium* was estimated to range from 6.1% in *B. indicum* to 26.5% in *B. saguini* (17), and while plasmids have been described in a few species, studies on the mobilome of these microorganisms reveal significant variability (25).

Endogenous plasmids have been identified in different *Bifidobacterium* species, including *B. longum, B. breve, B. asteroides, B. indicum, B. globosum, B. pseudocatenulatum*, and *B. bifidum* (25). Most of these plasmids are cryptic (26–32), except for one found in *B. bifidum* that encodes a bacteriocin (20, 25).

Various chromosomal regions in *Bifidobacterium* genomes are likely acquired through horizontal gene transfer (33). For instance, the genomes of *B. bifidum* contain between 4 and 54 insertion sequence elements, and the genome of *B. bifidum* 85B contains a predicted episome that includes genetic elements typically associated with conjugative plasmids (33).

The MGEs have also been examined in *B. breve* strains. This analysis revealed that *B. breve* JCM 7019 contains the highest number of MGEs in the genome (34). Additionally, a large conjugative plasmid, pMP7017, was identified in *B. breve* JCM7017 (35), and several homologous plasmids were subsequently found in other *B. longum* subsp. *longum* strains (36). The *B. breve* S27 genome appears to contain an integrated episome, designated S27-1, while an extrachromosomal plasmidial sequence was found in *B. breve* NCFB 2258, which is identical to the cryptic pCIBb1 plasmid from the same strain (26, 34).

Furthermore, studies of comparative genome hybridization have been conducted to investigate genomic variability within the *B. dentium* and *B. bifidum* species. In both species, the observed variable genomic regions include genes or gene clusters believed to encode restriction/modification systems, exopolysaccharide structures, pili, and various MGEs, such as insertion sequence elements, transposons, phages, and integrated plasmids (37, 38).

### Antibiotic resistance genes in *Bifidobacterium*

As probiotic microorganisms, one of the potential safety risks associated with *Bifidobacterium* members is the presence of antibiotic resistance genes (39), which could potentially be transferred to other bacteria and thereby represent a serious safety issue.

Although certain *Bifidobacterium* spp. exhibit antibiotic resistance phenotypes (40), most remain susceptible to antibiotics, such as beta-lactams, macrolides, vancomycin, chloramphenicol, and rifampicin (41). In most cases, the observed antibiotic resistance in *Bifidobacterium* is intrinsic, resulting from mutations in chromosomal genes (42, 43), and the likelihood of horizontal gene transfer is considered very low. Nonetheless, several antibiotic resistance genes have been identified within the *Bifidobacterium* genus, some of which are located on or near MGEs (39, 41, 44), raising concerns about their potential transfer to other bacteria.

The erythromycin resistance gene *erm*(X) is commonly found in *Bifidobacterium* within the transposon Tn5432 (44–46). Worryingly, the transfer of *erm*(X) by conjugation of a genomic island between *Bifidobacterium* strains was recently reported (47), highlighting a potential mechanism for horizontal gene dissemination.

## TA SYSTEMS

TA systems are small genetic modules that are widely distributed throughout bacterial and archaeal genomes (3, 48). They typically consist of a stable toxin and an unstable antitoxin and have attracted considerable interest due to their diverse functions in cellular physiology, stress response, and pathogenesis (49–51). Originally discovered on plasmids where they function to maintain plasmid stability through post-segregational killing (52), TA systems have since been identified in the chromosome of several bacteria, suggesting broader physiological functions (53, 54). These include the regulation of cell growth, biofilm formation, and MGE maintenance. Lately, phage inhibition has emerged as a prominent physiological function of TA systems, attracting increasing scientific interest (55, 56).

TA systems are currently classified into eight types (I–VIII) based on the nature and mechanism of the antitoxin, each with distinct regulatory and functional characteristics (49). Type II TA systems are among the most extensively studied (49, 57), and they consist of two protein components: a stable toxin that can disrupt essential cellular processes such as translation or DNA replication, and an antitoxin that directly binds and inhibits the toxin under normal conditions; upon exposure to stress or during specific physiological states, the protein complex is disrupted, freeing the toxin to exert its function. Overall, the antitoxin exhibits a modular structure, with the N-terminal domain functioning in transcriptional autoregulation through DNA binding, and the C-terminal domain responsible for neutralizing the toxin (58).

Several distinct families of type II toxins and antitoxins have been described and classified into superfamilies, based on sequence and/or structure homology (54). The two most studied are MazF/PemK/CcdB and HigB/RelE/ParE toxin superfamilies. MazF/PemK/CcdB toxins share a ribonuclease SH3 fold—even though their biological cellular targets are different—and have several different types of cognate antitoxins (54, 59). In particular, MazF is an RNA endonuclease that cleaves RNA at specific sequences independent of ribosome binding, thereby inhibiting translation (60), while MazE acts as its cognate antitoxin in most cases. HigB/RelE/ParE superfamily includes highly diverse toxins, with representatives such as RelE, ParE, HigB, YoeB, YafQ, and YhaV toxins, despite their distinct biological activities (61). For instance, RelE toxins are endoribonucleases that cleave mRNAs being actively translated by ribosomes as these transcripts enter the A site, while RelB, its most common cognate antitoxin, prevents its toxic activity under normal conditions (61–63). Hybrid TA systems mixing MazF-type toxins with RelB-type antitoxins, as well as RelE-type toxins combined with MazE-type antitoxins, have also been identified (64, 65), providing an evolutionary connection between both TA superfamilies.

While TA systems have been extensively studied in model organisms such as *Escherichia coli*, their roles in beneficial gut microbes like *Bifidobacterium* species remain largely unexplored, although they are attractive due to their potential impact on gut health and microbiota balance (66).

## OVERVIEW OF TA SYSTEMS IN *BIFIDOBACTERIUM*

The presence of TA system genes in bacteria of the genus *Bifidobacterium* was first reported in 2010 (67). In this study, TA systems were investigated across 27 species and subspecies of *Lactobacillus* and *Bifidobacterium*, revealing that TA systems were generally less abundant in the former, with the *relBE* system and its related genes, *yefM* and *dinJ*, being the most frequently identified. At that time, only two genomes were found to contain *mazEF* system genes. The authors also observed a strain-specific distribution of TA system genes—for instance, *B. adolescentis* ATCC 15703 carried the *mazEF* genes, which were absent in strain L232; conversely, L232 harbored the *relBE* genes, which were not found in ATCC 15703 (67). Three years later, the analysis was expanded to 36 sequenced genomes of bacteria of the *Bifidobacterium* genus, and it revealed the presence of 19 genes belonging to the MazEF and RelBE families in *B. adolescentis*, *B. bifidum*, and *B. longum* subsp. *longum* strains isolated from intestinal microbiota of astronauts and known sequenced genomes (68). On the contrary, TA genes of the MazEF and RelBE families were not detected in *B. angulatum*, *B. catenulatum*, or in two out of four *B. dentium* strains (68).

Since then, a huge number of *Bifidobacterium* genomes have become available, and several type II TA systems have been predicted through bioinformatic analyses; however, other types of TA systems have not yet been identified in this genus. Currently, the TA database TADB 3.0 (69)—which provides comprehensive information on types I–VIII TA systems—lists TA systems present in *B. longum*, (*n* = 265), *B. breve* (*n* = 123), *B. adolescentis* (*n* = 16), *B. catenulatum* (*n* = 11), *B. pseudolongum* (*n* = 7), *B*. sp. FKU (*n* = 6), *B. pseudocatenulatum* (*n* = 5), *B. thermophilum* (*n* = 4); three in *B*. sp. ESL0775, *B*. sp. ESL0769, *B*. sp. ESL0690, *B. pollorum*, *B. angulatum*, *B*. sp. ESL0728, *B. eulemuris*, and *B. subtile*; two in *B. scardovii*, *B. lemurum*, *B*. sp. ESL0800, *B*. sp. ESL0790, *B*. sp. ESL0682, and *B. asteroids*; and identified only one TA system in *B. ruminantium*, *B*. sp. ESL0764, *B*. sp. ESL0732, *B*. sp. ESL0704, *B*. sp. ESL0798, and *B. actinocoloniiforme* (see the supplemental material). Among these, only a limited number of TA systems have been experimentally validated, all of them from *B. longum* strains (70–73).

From these analyses, it is evident that most TA systems identified in *Bifidobacterium* belong to MazEF and RelBE families. Although a few representatives of YefM-YoeB, VapBC, and HigBA families could also be found (Table 1; also see the supplemental material).

**TABLE 1:** Main TA systems identified in *Bifidobacterium* and their putative biological roles

Antitoxin/toxin	Toxin activity/target	Species	Characteristics	Reference(s)
RelB/MazF	Ribosome-independent RNase	*B. longum*, *B. breve*, *B. infantis*, *B. animalis*, *B. adolescentis*, *B. bifidum*	Acid stress response; possible role in intestinal colonization	(67, 68, 70, 71, 73, 74)
DinJ/PemK	MazF-like toxin	*B. longum*, *B. breve*, *B. adolescentis,* other less represented strains	Identified but not functionally characterized	(67)
RelB/RelE	Ribosome-dependent RNase, it binds to 16S subunit	*B. longum*, *B. adolescentis, B. bifidum*	Osmotic stress response; regulation of cell growth	(68, 71, 72, 74)
YefM/YoeB	Ribosome-dependent RNase, it binds to 50S subunit	*B. pseudolongum*, *B. pseudocatenulatum*, *B. angulatum*, *B. subticle,* other less represented strains	Not characterized; associated to biofilm formation and virulence in other bacteria	(67, 68)
RelB/VapC	PIN-domain RNase	*B. longum*, other less represented strains	Identified but not functionally characterized	(68, 71, 75)
HicB/HicA	Ribosome-independent broad-spectrum RNase	*B. dentium*	Present in some strains, function undefined	(76)
HipB/HipA	Serine/threonine kinase that phosphorylates EF-Tu and inhibits translation; associated to bacterial persistence	*B. dentium*, pMP7017 (megaplasmid in *B. breve*)	Conserved in *B. dentium*; possible role in plasmid stability	(76, 77)
RelB/PumA	RelE-like RNase	*B. longum*, *B. breve*, *B. catenulatum*, *B. pseudolongum*, *B. angulatum*	Identified but not functionally characterized	Supplemental material

Interestingly, in *B. longum* subsp. *infantis* ATCC 15697, all the identified toxin components of the TA systems belong to different families—MazF, RelE, and VapC—while all the corresponding antitoxins are members of the RelB family (71).

A recent study explored the genetic variability of *B. dentium* and the presence of TA systems in 38 genomes (76). HipAB was the most conserved system among *B. dentium* genomes, identified in 25 out of 38 strains, followed by three RelBE systems identified in *B. dentium* BIOML-A1 and *B. dentium* BIOML-A2, and two HicAB systems identified in *B. dentium* 793B and *B. dentium* 2078B (76).

### MazF toxins

The first characterized type II TA system in *Bifidobacterium* corresponds to the *B. longum mazE_1_F_1_*^Bif^ genes (70). *B. longum* JDM301 BLJ_812 gene encodes a protein of 81 amino acids with homology to the RelB superfamily. On the other hand, BLJ_811 encodes a 107-amino acid protein belonging to the PemK/MazF interferase superfamily of toxins. Both genes form an operon with overlap of the open reading frames, characteristic in TA operons. Using *E. coli* as a heterologous expression host, it was demonstrated that the *mazE_1_F_1_*^Bif^ operon encodes a functional TA system: MazF_1_^Bif^ inhibited the growth of *E. coli*, and growth inhibition was alleviated by MazE_1_^Bif^ (70). Expression data suggested that MazE_1_F_1_^Bif^ may help *B. longum* JDM301 to cope with acid stress. In addition, Wei et al. (70) demonstrated that ClpP protease is also induced in the acid stress response of *B. longum* JDM301, and it degrades MazE_1_^Bif^ *in vivo*. Thereby, the authors speculated that, under acid stress, MazE_1_F_1_^Bif^ was activated through the hydrolysis of MazE_1_^Bif^ by ClpP in *B. longum* JDM301. Later, it was shown by an *in vivo* RNase assay that MazF_1_^Bif^ caused the degradation of the elongation factor Tu mRNAs (*tufA*^Bif^) and that this activity was alleviated by its cognate antitoxin, MazE_1_^Bif^ (73). In addition, MazF_1_^Bif^ was shown to physically interact with the noncognate antitoxin RelE^Bif^, or to MazE_2_^Bif^, which also alleviated the cell growth inhibition conferred by the MazF_1_^Bif^ toxin and counteracted the mRNA interferase activity of this toxin (73).

MazF toxins have also been identified and characterized in *B. longum* subsp. *infantis* ATCC 15697 (68, 71). This strain encodes three MazF toxins as part of *relB-mazF* modules (MazF1, MazF2, and MazF3), and a solitary *mazF* gene (MazF4), which are highly similar to orthologues from *B. longum* JDM301, *B. longum* subsp. *infantis* EK3, *B. breve* strains, and *B. longum* subsp. *infantis* 157F. All MazF toxins from *B. longum* subsp. *infantis* ATCC 15697 was highly toxic in *E. coli* in the absence of their cognate antitoxin genes, and their mRNA transcripts increased in the nutrient-depleted conditions of late stationary culture in the *B. longum* host (71).

It is noteworthy that all characterized MazF toxins from *Bifidobacterium* have a RelB antitoxin as their TA partner, forming hybrid TA systems composed of toxins and antitoxins from different families. Similar hybrid TA systems have also been described in *E. coli* (54, 64, 65) and seem to be common in *Bifidobacterium* (see the supplemental material).

### RelB and RelE proteins

A preliminary analysis of *Bifidobacterium* genomes indicated that *relBE* gene pairs were the most prevalent among the analyzed species (67). Further bioinformatics analysis conducted on 36 sequenced *Bifidobacterium* genomes (68), encompassing 11 species, revealed that the *relE* toxin gene was absent in approximately half of the examined strains. In contrast, the *relB* antitoxin gene was detected in all genomes except for five strains (68). Notably, *B. longum* strains exhibited the highest number of *relB* gene copies as well as the greatest degree of *relB* sequence polymorphism (68).

Experimental demonstration of the functionality of *Bifidobacterium* RelBE systems came from a study with *B. longum* subsp. *infantis* ATCC 15697 strain (71). Two *relB-relE* modules were identified in this strain (RelE1/RelB4 and RelE2/RelB1), in addition to *relB* antitoxin genes associated with *mazF* (RelB5–RelB7) and *vapC* toxin genes (RelB3), and an orphan *relB* gene (RelB2) (68, 71). The *relB3* gene was highly conserved among all the studied *B. longum* genomes but not in other species of this genus. RNase activity was demonstrated for one of those toxins (RelE2), as it was able to degrade the commercial MS2 RNA, and co-incubation with its cognate antitoxin (RelB1) inhibited this activity (71). The transcript levels of *relE1*, *relE2*, and the *relB2* genes were upregulated significantly in late stationary cultures of *B. longum* (71). The functionality of two *relBE* bicistronic operons from *B. longum* subsp. *infantis* ATCC 15697 was studied in the recipient *B. longum* subsp. *longum* NCC2705 strain, where they significantly decreased growth rate and diminished cell densities in the stationary phase (71).

A RelBE system was also identified in the chromosome of *B. longum* JDM301 (78) and then characterized (72). The loci encode two fully functional proteins; one is the RelE^Bif^ toxin that inhibits the growth of *E. coli*, and the other is RelB^Bif^ antitoxin that counteracts the inhibitory effect of its cognate toxin when expressed in *E. coli* (72). The expression of RelBE^Bif^ was increased during osmotic stress, and the toxin RelE^Bif^ could inhibit cell growth through mRNA degradation; furthermore, the activity of RelE^Bif^ can be antagonized by RelB^Bif^ (72).

In *B. longum* GT15, a RelB antitoxin was associated with a VapC toxin (75), similar to RelB3 from *B. longum* subsp. *infantis* ATCC 15697 (68, 71). This *relB* antitoxin gene is highly conserved, and it shares more than 90% identity with *relB* genes present in several *B. longum* genomes (68, 75). To our knowledge, the functionality and/or biological role of these hybrid TA systems have not been tested.

### YefM-YoeB TA systems

The YefM-YoeB TA system is widespread among plasmids and genomes of bacteria (54), with YoeB being the toxin, and YefM the antitoxin. YoeB is a homolog of the well-characterized RelE toxins, and they share structural similarity on the catalytic core, although low sequence identity (79, 80). Additionally, the two interact with the ribosome in distinct ways: YoeB associates with the 50S after ribosome dissociation (61, 62), while RelE interacts only with the 16S rRNA (47). Thus, YoeB and RelE inhibit translation by affecting initiation or elongation, respectively.

According to *in silico* data (see the supplemental material), several *Bifidobacterium* species harbor YefM-YoeB TA systems. Notably, in *B. pseudocatenulatum*, all identified TA loci belong exclusively to the *yefM-yoeB* family (supplemental material). However, to date, no *yefM-yoeB* TA system has been experimentally characterized in *Bifidobacterium*, and thus their functional roles remain unconfirmed.

### PumA toxins

PumA corresponds to a RelE-type toxin first identified in a pathogenicity island within the *Pseudomonas aeruginosa* plasmid pUM505, as part of a PumAB system (81). It was later characterized at the biochemical (82) and functional levels (83, 84). PumA was reported to exhibit 55% sequence identity with the RelE toxin from *Klebsiella pneumoniae*, and the *pumA* gene is 73% identical to an uncharacterized addiction module killer protein from *Pseudomonas putida* (83) that possesses only 22% identity to RelE from *Methanococcus jannaschii*, 18% to YoeB from *E. coli*, and 14% to RelE from *Pyrococcus horikoshii*. The low identity of PumA with RelE-like proteins is not surprising, as it has been reported that amino acid sequences of RelE-like proteins are quite divergent (61), even those originating from the same or closely related bacterial species.

Several *Bifidobacterium* species, including *B. longum*, *B. breve*, *B. catenulatum*, *B. pseudolongum*, *B. pullorum*, and *B. angulatum*, are predicted to encode PumA toxins, which are accompanied by canonical RelB antitoxins (see the supplemental material). Interestingly, according to these *in silico* data, in six out of seven *B. pseudolongum* strains, the only identified TA system corresponds to a PumA/RelB module. To date, no PumA toxin from *Bifidobacterium* has been functionally characterized, and it remains unclear whether these systems are transcriptionally active and biologically functional.

## BIOLOGICAL ROLES OF TA SYSTEMS IN *BIFIDOBACTERIUM*

The first study to demonstrate a physiological role for TA systems in gram-positive bacteria was published in 2005, and it demonstrated a role in acid tolerance of the MazEF and RelBE modules in *Streptococcus mutans* (85).

The physiological roles of type II TA systems in *Bifidobacterium* are still not fully understood; however, some authors have speculated that the presence of multiple RNase toxin genes allows these bacteria to regulate growth, survival, and metabolism over a broad range of environmental stresses (71).

### TA systems as acid stress response elements

Several studies have invoked a role for TA systems in responding to stress, although this has been increasingly questioned (49, 86). For instance, in the gram-positive bacteria *S. mutans*, a mutant lacking *mazF* and *relE* toxin genes was more resistant to acid killing than the parent or single mutants (85).

During the industrial manufacturing processes, passage through the digestive tract of the host, and storage, *Bifidobacterium*, and probiotics in general, encounter various stresses (19–23, 87). One of the major challenges they face is surviving the acid barrier of the stomach, after which viable cells can exert health benefits (21, 22). The acid tolerance of *Bifidobacterium* is mediated by adaptive cellular responses, such as alterations in cell membrane composition, upregulation of F0F1-ATPase enzymes, exopolysaccharide production, and shifts in metabolic pathways (21, 22, 88–90).

In the probiotic gram-positive acidolactic microorganisms *Lacticaseibacillus paracasei* and *L. rhamnosus* (formerly *Lactobacillus* [91]), two species component of the human gut microbiota, and extensively used as food additives, the DinJ-YafQ TA system has been shown to exhibit increased expression under nutritional, acidic, osmotic, and oxidative stress conditions, leading to a corresponding reduction in growth (92). Also, in the novel probiotic *Weisseria cibaria*, the HigBA TA system was shown to be activated when the bacteria was exposed to bile salt (93).

In *Bifidobacterium*, a role in acid stress response has been suggested for the MazE_1_F_1_^Bif^ system of *B. longum* JDM301, based on an increased transcription of *mazE_1_*^Bif^ in response to exposure to acid stress (pH = 2) for up to 2 h (70). As expression of *mazE_1_F_1_*^Bif^ was induced in response to acid stress, but did not increase under heat, cold, or nutritional stresses, the authors suggested that MazE_1_F_1_^Bif^ may help *B. longum* JDM301 to cope with acid stress. However, an increase in transcription was used as an indirect indicator of TA system activation via antitoxin degradation. Although it is now well established that upregulated transcription does not necessarily result in toxin activation (94).

In *B. longum* JDM301, transcription of *relBE*^Bif^ is induced under conditions of osmotic stress; in contrast, expression of *relB*^Bif^ did not appear to be upregulated in response to heat, cold, or nutritional stress (72).

While TA systems are not directly related to probiotic efficiency, they could rather play a role in their survival through their role in acid stress adaptation, which improves the ability to persist in host environments. We could hypothesize a similar role for TA systems in *B. dentium,* for which resistance to acidic environment is important for pathogenicity (24).

### TA systems as virulence/colonization factors

Contribution of TA systems to bacterial virulence is well documented (95) and these systems may play a role in pathogenesis and persistence under host-imposed stress conditions (95, 96).

Except for *B. dentium*, members of the *Bifidobacterium* genus are generally considered nonpathogenic. However, the probiotic efficacy of certain strains is likely linked to their capacity for effective colonization, a process in which the TA systems may have a role. For instance, in the probiotic *W. cibaria,* the activation of the HigBA TA system was involved in persister cells formation, which allows the bacteria to escape the bile acid stress and to improve viability within the gut (93).

All main TA systems identified in *Bifidobacterium* have been related to pathogenesis and persistence in the bacterial host. In the gram-positive bacterium *Staphylococcus aureus*, the YefM-YoeB system has been implicated in planktonic growth, extracellular-dependent biofilm formation, antibiotic tolerance, and virulence (97). Additionally, the MazEF system has been proposed to be essential for *S. aureus* pathogenesis (98) and to enhance biofilm-associated antibiotic tolerance, facilitating the transition from acute to chronic infections that are less virulent but more resistant to antibiotic eradication (99). To our knowledge, the PumAB system has been only characterized in the gram-negative bacteria *P. aeruginosa*, where it is known to increase the virulence, as it improves the ability of *P. aeruginosa* PAO1 to kill *C. elegans* (83). In addition, purified PumA protein decreased *C. elegans* viability, demonstrating that PumA could act as a bacterial virulence factor (83).

*Bifidobacterium* has been considered a biological targeting agent, being an innovative strategy for cancer therapy (100). The species *B. bifidum* and *B. dentium* in colorectal cancer patients exhibited a higher relative abundance than in the healthy control group (101). In contrast, the species of *B. breve* were less abundant in the colorectal cancer patient group compared to the healthy control group, indicating that *Bifidobacterium* might be an indicator of colorectal cancer (101). Indeed, using a mouse model of colorectal cancer, it was revealed that *B. breve* shows strain-specific anti-tumor effects by enhancing anti-tumor immunity (102).

Considering the strain-specific distribution of TA systems in *Bifidobacterium*, it warrants further research to reveal whether these genetic elements play a role in pathogenesis, persistence within the host, and/or the anti-tumor activities exhibited by certain strains.

### TA systems in *Bifidobacterium* MGEs

TA systems are recognized to be part of the accessory genome (59, 103), and to have a role in the maintenance of those mobile elements in the chromosome of the host bacteria (104, 105). In particular, plasmid-encoded TA systems were initially proposed to ensure the vertical inheritance of plasmids by eliminating cells devoid of the plasmid post-segregation (52).

Although plasmids are widespread among intestinal bacterial species, those of *Bifidobacterium* origin were thought to be uncommon, with only a few small cryptic plasmids identified in certain species (26–32). Nevertheless, the existence of large plasmids in the species *B. breve* was established with the discovery of the 190 kb conjugative megaplasmid pMP7017 in the host strain *B. breve* JCM7017 (35). This was followed by the identification of homologous plasmids to pMP7017 in other *B. longum* subsp. *longum* strains (36). Transcriptomic analysis of the pMP7017 megaplasmid revealed four transcriptionally active TA systems, belonging to three toxin superfamilies: AtaT/TacT, HipAB, and two systems from the ParE/RelE superfamily (77). However, the biological functions of these systems remain unclear.

On the other hand, the PumAB system confers plasmid stability to *P. aeruginosa* (83). Notably, in *Bifidobacterium*, certain PumA toxins are encoded within putative MGEs (see the supplemental material), suggesting a potential role for this TA system in MGE maintenance.

### TAs as markers for metagenome analyses

Accurate characterization of the gut microbiome is essential, as it significantly influences health assessment evaluation, personalized treatment strategies, and disease prevention (106). The impact of the gut microbiome on the host varies among individuals and is determined not only by differences at the phylum or species level, but also by variations at the strain level (107, 108). However, current methods for strain-level characterization are still not adequate, and the human microbiota is typically analyzed at the family or genus level, with limited resolution at the species and strain levels.

In *Bifidobacterium*, the distribution of toxin and antitoxin genes was identified to be species- and strain-specific (68). Analysis of gene polymorphism (presence of nucleotide substitutions and deletions in detected genes) in *mazEF* and *relBE* TA genes in *Bifidobacterium* isolated from intestinal microbiota revealed that the observed polymorphism of antitoxin *relB* genes in *B. longum* can be used as criteria for the strain identification and isolation of polymorphic subtypes (68). Later, it was shown that genes of type II TA systems can be used as functional markers for computer-assisted species and strains characterization of *Lactobacillus* and *Bifidobacterium* in the human gut microbiota (74), which led the authors to develop the TAGMA (toxin antitoxin genes for metagenome analyses), a software tool based on polymorphism in TA genes that was tested on five gut metagenomic samples. The results demonstrated that TAGMA could reliably identify *Lactobacillus* and *Bifidobacterium* species—and in some cases, even distinguish individual strains or specific groups of strains within the metagenomes (74). Subsequently, the analysis was extended to include a larger set of TA proteins and genes, as well as a broader range of bacterial species (109). The results reinforced the potential of TA systems as an effective tool for computer-assisted species and strain-level characterization of the human gastrointestinal microbiota.

## WHY DOES *BIFIDOBACTERIUM* ENCODE FEW TA SYSTEMS?

Comparative genomic studies have shown that genomes of free-living bacteria usually encode many TA system homologs (103, 110), and that the presence of TA systems is significantly associated with the pathogenicity of bacteria (51). For example, *M. tuberculosis* encodes more than 100 TA systems (111), of which at least 30 are functional (112, 113); whereas its nonpathogenic counterpart, *M. smegmatis*, has only three functional TA systems (112). In *Bifidobacterium*, different ecological relationships between these bacteria and their host can be developed, ranging from opportunistic pathogenic interactions (e.g., in the case of *B. dentium*) to a commensal or even health-promoting relationship (e.g., in the case of *B. bifidum* and *B. breve* species). Thus, due to the limited information available regarding the presence of TA systems in *B. dentium*, it is plausible to speculate that this species encodes a distinct number and diversity of TA systems compared to its nonpathogenic counterparts. In addition, we cannot rule out the possibility that *Bifidobacterium* encode TA systems other than type II, such as type I or types III–VIII, different from those currently included in the TA database TADB 3.0.

In the seminal genome comparative study between gram-positive lactic acid bacteria of the genera *Lactobacillus* and *Bifidobacterium* (67), the authors hypothesized that the relative scarcity of the TA system genes in *Bifidobacterium* genomes, compared to *Lactobacillus*, could result from different tempos of evolution in the same ecological niche (intestines) and different adaptation to the conditions of this niche. This observation clearly warrants further investigation.

## CONCLUSIONS

*Bifidobacterium* is among the most dominant and well-recognized health-promoting probiotic microorganisms. Given the involvement of TA systems in bacterial adaptation to fluctuating environmental conditions, their study in *Bifidobacterium* represents a particularly relevant area of research, as it may offer valuable insights into their ecological roles and unveil novel applications in probiotics and gut health.

To date, most studies on TA systems in *Bifidobacterium* have relied primarily on bioinformatic and transcriptional analyses. However, functional studies are essential to elucidate the specific mechanisms and physiological roles of these systems across different *Bifidobacterium* species. For instance, the TA systems of *B. dentium* are of particular interest due to the pathogenic nature of this species, and their study may provide valuable insights into their biological roles and potential involvement in virulence and stress adaptation. Further, it would be particularly interesting to study the YefM-YoeB and PumA/RelB TA systems, as they are common but have not yet been characterized in this genus. Moreover, understanding why certain TA systems are exclusively associated with specific species or even individual strains could provide valuable insights into their functional significance and evolutionary roles.

Advancing this line of research will deepen our understanding of TA systems and their broader implications for host health and disease.

## SUPPLEMENTAL MATERIAL

Supplemental material aem.01934-25-s0001.xlsx TA systems encoded by *Bifidobacterium* according to TADB.

ASM does not own the copyrights to Supplemental Material that may be linked to, or accessed through, an article. The authors have granted ASM a non-exclusive, world-wide license to publish the Supplemental Material files. Please contact the corresponding author directly for reuse.
